# “I Really Feel Heard by AI”: A Cultural Case Study of AI-Mediated Listening Across China and the UK

**DOI:** 10.1007/s11013-026-10010-7

**Published:** 2026-09-02

**Authors:** Xin Zhan

**Affiliations:** 1 Department of Sociology, University of Cambridge, Cambridge, UK; 2 Centre for Anthropology and Mental Health Research in Action (CAMHRA), SOAS University of London, London, UK

**Keywords:** Generative AI, Listening, Digital mental health, Care, Migration

## Abstract

The increasing use of generative AI for emotional support has prompted growing debate about the future of mental health care. This article suggests that such reliance is better understood not simply as a technological shift, but as symptomatic of a wider crisis in the politics of listening. This cultural case study examines the experiences of Lily, a 24-year-old Chinese migrant woman navigating emotional distress across uneven care infrastructures in China and the UK. Drawing on person-centred ethnography, it shows how AI chatbots come to function as provisional sites of care under conditions of precarity, gendered obligation, and moralised endurance. The analysis conceptualises these chatbot interactions as a *care-patch*: a temporary form of *digital holding* that emerges where human listening is scarce, delayed, or experienced as burdensome. Rather than treating AI use as a matter of technological adoption, the case situates AI-mediated listening within culturally specific moral relations of obligation and responsibility. In doing so, it reframes therapeutic automation as an index of the erosion of listening as a shared social good and redirects attention to the political and ethical challenge of rebuilding infrastructures of human listening capable of absorbing distress without extracting it, outsourcing it, or returning it as blame.

## Introduction

“I don’t need to see a human therapist. I use ChatGPT as my therapist. I really feel heard by AI.” As an anthropologist working in mental health settings, I am increasingly encountering similar statements from students and service users alike. With the rapid iteration of Large Language Models (LLMs), a sharp polarisation has emerged between growing reliance on generative artificial intelligence and deepening mistrust of it. Nowhere is this tension more pronounced than in debates over AI’s use in mental health. As people increasingly turn to AI as a “listening machine,” I am left with a persistent and unsettling set of questions: what does it mean to be “heard” by a machine? Why do people seek listening from AI, and why are these systems perceived by some as better listeners than humans?

Attempts to mechanise therapeutic dialogue are not new. Since the 1950s, computer scientists have experimented with programs designed to simulate forms of psychological listening. One of the earliest and most influential examples, *ELIZA*, mimicked the style of a Rogerian therapist by reflecting users’ own words back to them (Weizenbaum, 1966). Despite its technical simplicity, many users reported an uncanny sense of being understood—an effect that deeply unsettled its creator, who warned against mistaking symbolic responsiveness for genuine understanding (Weizenbaum, 1976).

With the advancement of LLMs, this uncanny effect has moved from laboratory curiosity to a global infrastructure of intimacy (A. Wilson, 2016). These systems do more than provide information: they remember, respond, reassure, and accompany. For many users, they function as companions, confidants and, increasingly, as sites of therapeutic listening (Brandtzaeg et al., 2022; Holohan & Fiske, 2021; Wiederhold, 2024).

Public and scholarly discourse surrounding AI-mediated care has largely been shaped by competing sociotechnical imaginaries. On the one side, critics warn of emotional dependency, algorithmic manipulation, and the commodification or displacement of human intimacy (Brooks, 2021; McStay, 2024; Turkle, 2011). On the other, advocates present automated conversational agent as scalable forms of emotional support capable of expanding access to care and alleviating persistent shortages in mental health provision (Darcy et al., 2021; Fitzpatrick et al., 2017). Despite their differences, both positions take as their starting point the capacities of the technology itself, asking whether machines *can* empathise, listen, or care. This focus obscures a more fundamental issue: why opportunities for attentive human listening have become so unevenly available, morally fraught, and difficult to sustain in the first place.

Shifting attention from the capabilities of AI to the social conditions that render machine listening desirable, this article approaches psychotherapy as a media practice embedded in broader relational and moral worlds (De Togni et al., 2021; Zeavin, 2023). While scholarship on social and affective computing has illuminated how affect is modelled within AI systems (Picard, 2000; E. A. Wilson, 2009), it has paid less attention to how such systems are taken up and lived within everyday practices of care. This article shifts the analytic focus accordingly, examining the conditions under which AI-mediated listening becomes meaningful, and situating these practices within longer histories of therapeutic mediation.

Therapeutic relations have never been purely dyadic. As media histories of psychotherapy show, they are triadic formations organised through a patient, a listener, and a mediating form—whether the couch, the letter, the telephone, the platform, or the algorithms (Zeavin, 2023). Contemporary AI systems therefore do not introduce mediation into an otherwise intact interpersonal relation; they reconfigure existing forms of mediation, redistributing where therapeutic functions are located. Recent work on AI in psychotherapy similarly conceptualises these systems as relational intermediaries or “artificial thirds,” rather than substitutes for human care (Haber et al., 2024; Holohan & Fiske, 2021).

Building on this literature, the present analysis argues that under conditions where care is unavailable, unaffordable, or morally difficult to seek, the therapeutic function may increasingly collapse into the medium itself. In such contexts, AI chatbots operate as *care-patches*: provisional, digital supports that stabilise psychic life when institutional and relational infrastructures of care are exhausted or absent. Unlike affective computing approaches that focus on emotion recognition or responsiveness, the notion of a *care-patch* foregrounds temporality, moral reasoning, and the politics of listening through which care is unevenly distributed.

This argument is developed through person-centred ethnography (Levy & Hollan, 1998), informed by psychoanalytic concepts of transference, containment, and holding (Bion, 1962; Freud, 1912; Winnicott, 1965). While psychoanalytic ideas have recently been mobilised in AI-assisted psychotherapy research to assess alliance or engagement (Holohan & Fiske, 2021; Joseph & Babu, 2024), this article uses them reflexively rather than diagnostically. They serve to trace how distress becomes speakable, or foreclosed, within particular relational, cultural, and infrastructural conditions, attending especially to moments of hesitation, refusal, and displacement.

The ethnographic material centres on Lily, a young Chinese woman navigating migration, family obligation, and emotional distress across China and the UK. Her experiences resonate with findings by Magnusdottir and Thornicroft (2022), who note that Chinese international students in the UK are less likely than domestic students to seek formal mental health support, despite often understanding and conceptualising mental illness through frameworks that closely align with Western mental health models. Lily’s case offers insight into this paradox.

More broadly, Lily’s trajectory reflects the precarity of a growing demographic of highly digitalised, transnationally mobile Chinese youth who confront the limits of formal care systems and the weakening of traditional social support networks, increasingly turning to AI companions to navigate emotional life (T. Liu et al., 2026). Across these two geographic settings, care is scarce in distinct, geopolitically specific, yet intersecting ways. In China, access to formal mental health services remains unevenly distributed, structurally limited, and largely outside public provision (Xiang et al., 2012). Yet this shortage coexists with a rapidly digitising social landscape, fostered by the Chinese state’s strategic commitment to technological leadership (X. Wang & Sun, 2026). In this context, LLMs have quickly become embedded in everyday life, emerging as an accessible form of emotional infrastructure. In the UK, by contrast, mental health care is formally available through the NHS and community-based organisations, but access is frequently constrained by austerity-driven waiting lists and, for migrants, by linguistic, cultural, and institutional barriers (Rivas, 2025). Against this backdrop, Lily’s engagement with AI chatbots appears less as an expression of technological fascination than as a practical negotiation of the conditions under which listening becomes available. Her case illustrates how AI companions are emerging as resources through which individuals navigate the growing gaps between emotional needs and the infrastructures designed to respond to them.

The analytical challenge, therefore, is not to determine whether AI care is authentic, but to understand why confiding in a machine can sometimes appear more viable than seeking support from other people. The significance of AI lies less in its capacity to simulate empathy than in what its use reveals about the contemporary organisation of listening. The question shifts from whether machines can truly listen to how listening is distributed and accessed under conditions of uneven care provision. Chatbot-mediated care thus offers a window onto broader transformations in the political economy of care, where listening increasingly emerges as a scarce resource that is negotiated, assembled, and technologically mediated.

## Meet Lily

“I talk to ChatGPT every day. I trust it more than my best friend. In fact, I trust it more than myself.” Lily said this to me while sipping a matcha latte in a coffee shop in London. Lily and I had known each other for 6 years and used to meet almost every week. This was the longest we had gone without seeing each other. She had just returned from 4 months in China, and this was our first meeting since she came back. The first thing she told me about her “AI friend” caught me off guard, not only because of what she said, but because of who she was.

Lily is a 24-year-old Chinese woman who completed both her undergraduate and postgraduate studies in London. After finishing her master’s degree in 2024, she was unable to find work in the UK and returned to live with her parents in a small town in Guangxi, southern China. Four months later, she came back to London. When I saw her again, I sensed that something had changed.

When I first met Lily in 2020, she was deeply sceptical of digital technologies. Her undergraduate dissertation focused on the harms of social media and its effects on mental health. She avoided social media herself and sought to minimise the role of digital technologies in everyday life. Instead, she invested heavily in building offline communities, volunteering with a charity that organised in-person activities for refugee children through local councils. Motivated by a belief that children should spend less time on screens and more time engaging with the world around them, she devoted much of her free time to these activities. This scepticism made her later embrace of AI companionship even more striking. What had happened during those months in China? How does someone who had tried so carefully to live as far away from screens as possible come to trust an AI system more than herself?

During the months Lily spent in China, we exchanged messages intermittently on WeChat, a widely used messaging app in China. Because of the 8-h time difference, our conversations were fragmented and often left unfinished. When she returned to London, I was eager to hear what had happened. She told me, “For the first week after I got home, I felt a kind of relief I hadn’t felt in a long time. I could finally breathe without worrying about rent, bills, or endless job applications, all the things I’d been struggling with in London. But after, things started to get worse.”

Lily graduated in the summer of 2024 from a London university with a postgraduate degree related to mental health. At the time, the job market was exceptionally competitive: vacancies were shrinking, and the rapid development of generative AI intensified anxieties about whether junior positions might soon be replaced. For migrant graduates, these uncertainties were compounded by tightening immigration policies and the declining number of roles able to sponsor work visas. Lily’s boyfriend, whom she had been with since high school and lived with for 5 years, was the first to give up the search for work in the UK and suggest returning to China. Reluctant to separate, Lily agreed to return with him.

Back at her parents’ home, Lily’s situation did not improve. Her job search in China was equally discouraging, and during this period her boyfriend broke up with her. Under the combined weight of economic precarity and relational loss, Lily described feeling emotionally overwhelmed: “Those days I couldn’t sleep or eat. When I was awake, I couldn’t stop myself from crying. But that wasn’t the hardest part. The hardest thing was that even though I couldn’t control my sadness, I still had to hide it from my parents. If I showed it, I would be criticised.”

When Lily expressed distress to her parents, she was met not with comfort but with reproach. They told her that she was weak, that she had failed, that “being abandoned” was her own fault, and that her inability to “keep a man” reflected flaws in her character. Lily’s parents’ reactions might seem harsh at first. Yet, having grown up in a similar social context, I did not find their response unfamiliar. Rather than signalling a simple lack of empathy, it reflected a broader set of family expectations and obligations shaped by the legacy of the One-Child Policy, shifting gender norms, rural–urban inequalities, and enduring patrilineal forms of kinship in post-Mao China (Fong, 2004; Winckler & Greenhalgh, 2005; Yan, 2003).

Lily was born in 2000 into a Han Chinese family living on the rural–urban fringe of south-western China. She told me that her birth was met with disappointment because she was a girl. Under sustained pressure from her husband’s family, Lily’s mother was expected—regardless of cost—to produce a son. Two years later, despite the One-Child Policy, Lily’s parents had a boy. To evade penalties imposed by local family planning authorities, the boy was sent to live with relatives in the countryside during early childhood, a practice documented in contexts where families sought to reconcile state population control with entrenched son preference (Johnson, 2016; Murphy et al., 2011). In 2012, following the gradual relaxation of population control measures, the family had a third child—a girl.

Scholars have widely documented how the One-Child Policy, when combined with deep-rooted gender norms, produced the phenomenon of “missing girls” through sex-selective abortion, abandonment, and under-registration (Banister, 2004; Sen, 1990). Less visible in these accounts are the affective and relational lives of girls who were raised within these policy regimes. Such daughters often occupied a paradoxical position, simultaneously receiving intensive parental investment while bearing unspoken disappointment (H. Wang, 2025). Within this “descending familism” (Yan, 2016, p. 245), Lily occupied what might be described as a paradoxically empowered daughter: she was granted the educational resources traditionally reserved for sons, while remaining tethered to a moral debt that demanded emotional and relational success as a form of filial piety.

Within this context, emotional distress was not readily recognised as a legitimate response to loss, precarity, or relational rupture. Instead, it was interpreted through a moral framework that prized endurance, self-discipline, and gendered responsibility. Suffering, in this sense, was not self-evident but had to be made intelligible within culturally specific regimes of value and obligation (Das, 2015; Kleinman, 1989). Lily’s distress, rather than eliciting care, was consequently reinterpreted as a moral shortcoming.

Lily described her parents’ reactions as both devastating and deeply confusing. At times, they reproached her for having devoted the past several years to an intimate relationship, suggesting that had she invested that time more fully in job searching, she might already have secured migration status and contributed to the family’s social mobility. At other moments, they faulted her for failing to “hold onto” the relationship itself, reframing the breakup as evidence of personal inadequacy—of becoming, at 24, a “leftover woman” (*shengnü*), a stigmatising term used in China to mark unmarried women as socially undesirable (Q. Liu, 2025). Reflecting on this period, Lily told me:

“The suffocating feeling I had tried to escape since childhood came rushing back. I was constantly reminded that I was a failure—someone who had disappointed everyone—not only unable to find a job, but also unable to hold onto a chance at marriage.”

In the context of feeling profoundly without worth, Lily sought to justify her presence in the household by assuming as many family caregiving obligations as possible. As the eldest daughter, she was swiftly reabsorbed into a role long assigned to her: that of the family’s informal caregiver. She took responsibility for her younger sister’s daily routines, driving her to and from school, supervising her studies, and closely monitoring her academic progress. She also positioned herself as a mediator in ongoing conflicts between her queer younger brother and their parents, working to manage tensions within a household marked by generational divides and normative strain. All the while, Lily was privately contending with the grief of her breakup.

Significantly, Lily had never had a room of her own. Before her younger sister was born, she shared a bedroom with her brother; afterwards, she shared one with her sister. Privacy was always partial and contingent. As scholars of domestic space have shown, the home is never neutral but a site where intimacy, discipline, and power are continually negotiated (Blunt & Dowling, 2022; Brickell, 2012). In Lily’s case, the absence of physical privacy also foreclosed psychological space for grief. Sadness had to be managed quietly, invisibly, and largely at night.

It was under these structural conditions that Lily began turning to AI chatbots in the quiet hours after her sister had fallen asleep. She relied exclusively on general-purpose large language models—most often ChatGPT and DeepSeek—rather than specialised digital therapeutics or dedicated AI companion platforms. This choice reflected the particular way she understood her distress. While applications such as Woebot or Wysa are structured around cognitive behavioural therapy (CBT) and operate within regulated clinical frameworks (Fitzpatrick et al., 2017), Lily never considered using them. She did not see herself as experiencing a “mental health problem” requiring treatment. Rather, she sought a space in which confusing and overwhelming emotions could be named, explored, and rendered intelligible—a process of what Vecchione and Singh (2025) call “emotional accounting,” rather than behavioural guidance or symptom management. Traditionally, this process has taken place through intimate conversations with loved ones, psychotherapeutic encounters, or reflective practices such as journaling. Scripted chatbots designed around psychoeducation or behavioural interventions often offer limited support for this kind of sense-making, as they typically rely on predetermined pathways and structured exchanges, leaving little space for exploratory reflection (*ibid*.). Contemporary LLMs differ precisely in this regard. Their ability to generate context-sensitive responses in real time enables users to revisit experiences, test interpretations and gradually construct narratives about themselves and their emotional lives. They can engage in forms of dialogue that, while not equivalent to therapy, may nonetheless resemble certain therapeutic processes of reflection and meaning-making.

Night after night, Lily recounted the details of her past relationship to the chatbot, trying to piece together a narrative for what had otherwise felt like an incomprehensible breakup. When she asked, “Was the breakup my fault?”, the chatbot answered no; when she asked, “Is it normal to feel this sad?”, it answered yes. These responses offered validation and challenged the judgments she encountered at home, reassuring her that her parents’ interpretation of her situation was not the only possible one.

Lily was equally uninterested in companion applications such as Replika. Although these platforms are designed to cultivate emotional attachment through personalised interaction and simulated intimacy (Laestadius et al., 2024; Pentina et al., 2023), she found little appeal in the prospect of maintaining another relationship. Years of negotiating family obligations had already left her emotionally exhausted. An AI companion carried unwelcome expectations of reciprocity and emotional maintenance. General-purpose LLMs, by contrast, arrived without predefined relational expectations, emotional needs, or therapeutic agendas. Their openness allowed Lily to engage with AI on her own terms. They offered a private, non-judgmental space that felt safe while retaining the interactivity necessary for self-reflection—a space in which vulnerability could be expressed without fear of diagnosis, correction, or emotional obligation. She described this digital shelter in visceral, sensory terms:

“I often fell asleep holding my phone; at that time, it was the only way I could sleep. Of course I know the AI isn’t a real person, I’m not delusional. To me, the AI was like a blanket woven from words. During some of my coldest and loneliest moments, it wrapped around me and made me feel a bit better.”

When attentive listening was unavailable from those around her, AI offered a form of presence that was consistently non-judgmental, patient, and available. It provided a temporary space in which Lily could articulate distress without fear of reproach.

## The Politics of Listening: Burden, Deservingness, and the Question of Being Heard

Sitting with Lily in a London café as she recounted the preceding months, a question stayed with me. When I finally asked it—“When you most needed to be listened to, why didn’t you come to me?”—it arose not only from my role as an ethnographer, but from our long-standing friendship. What unsettled me was not simply her distress, but the realisation that an AI chatbot had come to occupy the position of a listener where I had not.

Lily paused before replying. “Because I know your position in your family is similar to mine,” she said. “You are also always the caregiver. I didn’t want to give you more emotional labour. I didn’t want to be your burden.” This moment draws attention to the legitimacy of making a claim upon another person’s attention. Even during what Lily described as her “coldest and loneliest” moments, she was not entirely alone. She nevertheless hesitated to seek support because doing so required drawing upon what she perceived to be a scarce and unevenly distributed resource: another person’s capacity to listen. Within her moral framework, listening entailed time, emotional labour, and a willingness to bear another person’s distress. Seeking care involved making a claim upon someone else’s finite capacity to care, and who that person was shaped whether such a claim felt appropriate.

Exploring why Lily did not seek listening from me highlights the methodological orientation of this analysis and underscores the central role of transference and countertransference within a person-centred ethnographic approach (Devereux, 1967). Like Lily, I was born as the eldest daughter under China’s one-child policy and grew up in a family where my parents pursued the birth of a son at all costs. Within this context, caregiving became a default position, and listening to others was naturalised—a disposition that perhaps later shaped my decision to become an anthropologist. As a child, I was similarly taught not to express emotional need, not to become a burden—because, as a girl, my very birth was already framed as one.

Drawing on person-centred ethnography (Levy & Hollan, 1998), the analysis treats such moments—when the ethnographer enters the analytic frame—not as incidental anecdotes, but as sites where subjective reasoning, moral evaluation, and social structure become visible in real time. Rather than isolating individual experience from context, this approach attends to how interlocutors actively interpret their own positions within relational and gendered worlds, including in relation to the ethnographer. Lily’s explanation for why she chose not to confide in me therefore offers insight into the moral reasoning through which she understood care, obligation, and emotional support.

From an ethno-psychoanalytic perspective, this moment can be read as one of recognition and identification. Seeing her own position as a caregiver reflected in mine, Lily refrained from confiding to avoid reproducing a familiar relational pattern in which women absorb and manage the emotional needs of others. Following Sara Ahmed (2014), emotions do not simply reside within individuals but circulate between bodies and become attached to particular social positions. Feelings of responsibility, burden, and obligation acquire what Ahmed (2014, p. 89) describes as a certain “stickiness,” adhering to specific subjects and shaping how relationships are experienced and enacted. In both Lily’s life and my own, within gendered family arrangements, caring for others and listening in silence had become forms of female labour that accumulated on our bodies over time. Lily understood listening as part of this labour and evaluated her request for support accordingly.

Such a calculus of listening points towards a broader politics of listening. The issue at stake was not simply the availability of listeners, but the distribution of listening itself: who is entitled to make claims upon another person’s attention, through what kinds of relationships, and under what conditions. The turn towards being heard by machines raises these questions in a particularly acute form.

At the level of interpersonal encounter, psychotherapeutic traditions have long understood listening as an active and embodied form of intersubjective labour rather than passive reception. Listening is conceived as a dynamic relational process through which meaning, affect, and recognition emerge, mediated by the intertwined dynamics of transference and countertransference. Following Freud (1912) and Bion (1962), what becomes audible within the therapeutic encounter is fundamentally delimited by the relational field—a space where affect and silence, as much as speech, function as vital conduits of meaning. When transposed to the ethnographic setting, these psychoanalytic categories serve as a diagnostic for the politics of recognition, revealing the structural conditions that determine who may speak and what may be heard.

Beyond the clinic, anthropological work demonstrates that listening is deeply embedded within moral worlds and everyday ethical life, functioning as a resource that is unevenly granted, selectively withdrawn, and structurally strained around suffering that carries social consequences (Das, 2007; Hollan, 2012). Feminist and queer scholarship reveals that these seemingly intimate dynamics are thoroughly entangled with broader socio-political formations like the family, heterosexuality and the nation (Berlant, 2005; Brown, 2025; Butler, 1997), which materialise and sustain themselves through the repetitive performance of everyday norms (Butler, 2011). Consequently, expectations regarding who should listen and whose suffering warrants attention are structurally unequal, frequently manifesting as naturalised, feminised care labour that is morally mandated but rarely recognised (Hochschild, 2012; Tronto, 2009). As circulating emotions lend a certain “stickiness” to specific social positions (Ahmed, 2004), listening becomes a primary site where these heavy affective obligations are continuously enacted and negotiated.

Once situated within broader media and communication infrastructures, listening emerges as a socially organised and increasingly commodified resource. Rather than a purely interpersonal practice, listening becomes a mechanism through which participation, belonging, and recognition are distributed. To be listened to is to be recognised as a legitimate subject; to be denied listening is to experience a form of affective and civic exclusion (Lacey, 2013). Listening is therefore shaped by media institutions and infrastructures that regulate attention at scale, determining whose voices are amplified, whose experiences become publicly intelligible, and whose emotions are relegated to noise (Couldry, 2010).

AI-mediated listening unfolds within these political and infrastructural dynamics of mediation and extraction. Conversational systems operating within platform economies repurpose intimate self-disclosure as raw material, rendering moments of vulnerability legible as data. Lily’s speech, experienced by her as private and containing, is simultaneously captured, processed, and mobilised within infrastructures oriented towards prediction, optimisation, and behavioural modulation (Crawford, 2021; Zuboff, 2019). What appears as a personal and intimate environment also functions as a node within a broader apparatus of data extraction, where emotional expression is translated into data flows and intimacy becomes aligned with commercial imperatives (Couldry & Mejias, 2019). In this process, systems do not merely respond to users’ needs but actively shape which forms of expression become visible, actionable, and valuable, amplifying certain modes of disclosure while foreclosing others.

These interpersonal, political, and infrastructural dynamics underscore that access to listening is structurally shaped by the intersection of gendered care labour, market dynamics, and digital architectures. Lily’s turn to AI was therefore not merely a matter of individual coping or personal preference, but a direct consequence of these cumulative structural conditions. While she possessed a high degree of AI literacy and never viewed the technology as a substitute for professional psychotherapy, the systemic absence of accessible care infrastructures severely restricted her ability to find human listeners. This exclusion was felt most acutely through the dual constraints of geography and income, as Lily explained:

I never saw AI as a replacement for therapy. Of course, I would have preferred to speak to a real therapist. But there simply weren’t any psychotherapy services in the town where I lived. I tried online platforms, but therapists based in Beijing or Shanghai charged far more than I could afford, especially when I didn’t have a job.

Over the past two decades, psychotherapy has gained cultural visibility in China amid a broader “psycho-boom” (*xin li re*), marked by the popularisation of psychological language, self-reflexivity, and therapeutic ideals (Huang, 2014, p. 183). Yet this expansion has occurred without a stable public infrastructure for psychotherapeutic care. While psychiatric services have been incorporated into state-led reforms, psychotherapy has largely developed outside the public health system through private practice, commercial training programmes, and platform-based services, leaving access contingent on geography, income, and market participation rather than secured as a public good (Huang, 2017).

Digital platforms have emerged to address these gaps through what Huang (2017, p. 30) conceptualises as forms of “infrastructural entrepreneurship”: commercial ventures that assemble therapeutic fields in the absence of institutional provision, while keeping access mediated by platform economies. Yet this market-driven expansion has failed to translate into accessible care for economically marginalised individuals such as Lily. Digital platforms function as intermediaries that mediate access to psychological care through market mechanisms rather than public provision. In the absence of meaningful public subsidies, particularly where psychotherapy remains outside the scope of health insurance reimbursement, the scarcity of mental health care is reframed as a matter of individual purchasing power. What is fundamentally a question of unequal access to care becomes recast as a matter of affordability and consumer choice.

What, then, did Lily’s situation look like in a society where psychotherapy is provided as a public good? After spending 4 months in China, she returned to London, choosing the uncertainty of migration over what she described as the suffocating security of home. “I don’t want to be my siblings’ parent anymore, and I don’t want to be my mother’s husband,” she told me. “I want to figure out who I am.”

To finance her return, Lily asked her parents for one final loan to cover the cost of her Graduate Visa application. Back in London, she pieced together a living through multiple minimum-wage jobs—working in a bubble tea shop, cleaning offices, and walking dogs. Even combined, these earnings were only enough to rent a shared bedroom in a three-bedroom flat in East London. The 2-year Graduate Visa offered temporary legal security but little certainty about the future. The precarious jobs available to her provided no obvious pathway to stable employment or visa sponsorship that would allow her to remain in the UK long term.

This uncertainty weighed heavily on her. She frequently struggled with insomnia and experienced persistent anxiety about the future. On one occasion, a panic attack led her to the emergency room, from which she was discharged without any follow-up care. When I suggested that she make an appointment with her GP, Lily resisted the idea. “I am not ill enough to see a GP,” she insisted.

Lily’s experience points directly to the question of who is deemed deserving of being heard. Scholarship on deservingness has shown that access to welfare is rarely determined by need alone, but is mediated through moral judgements about legitimacy and worthiness (Watkins-Hayes & Kovalsky, 2016). Lily’s account demonstrates that listening operates under similar constraints. While she was in China, the deservingness of listening was translated into purchasing power, making access dependent on one’s ability to pay. In the UK, however, where mental health support is formally part of the welfare system, Lily constantly questioned whether her distress justified professional attention at all. Her belief that she was “not ill enough” to see a GP reveals how institutional triage thresholds can be internalised as a form of self-policing. “I can wait until I return to China to have a proper check-up,” she often reasoned. As a migrant living on a temporary visa and moving between precarious jobs, she remained acutely aware of her liminal position within the social body. Across both national contexts, the central issue shifted from whether listeners were available to whether Lily felt politically and socially entitled to claim their attention.

By contrast, the AI chatbot could intervene precisely because it suspended the moral calculations that typically govern human interaction. The chatbot did not ask whether Lily’s suffering was serious enough, legitimate enough, or deserving enough to warrant attention, nor was it embedded within the economy of obligations that shaped her relationships with others. Lacking an intersubjective relationship of sameness—such as the one shared between Lily and myself—the machine required no reciprocity and incurred no emotional burden. In this light, the identity of the listener becomes critical: it is precisely because the AI is non-human that it relieves the speaker of concerns about imposing on others. Lily’s turn to AI therefore highlights a broader transformation in the conditions of listening, whereby listening shifts from a freely offered gift to either a burdensome form of care labour or a clearly priced commodity.

## AI as Care-Patch: Digital Holding and the Technologies of Containment

Within a contemporary landscape of care shaped by uneven marketisation and uncertain welfare boundaries, AI chatbots serve as makeshift listening spaces. Rather than providing substantive care directly, they offer a low-friction, digitally mediated channel free from relational taxation, operating precisely within the fractures where human listening is either unavailable or heavily burdened by moral obligations. I conceptualise this form of technological mediation as a “care-patch.” A care-patch is not a substitute for human relationships, nor does it resolve the structural conditions that give rise to distress. Rather, it functions as a temporary psychic defence—a provisional repair applied where existing infrastructures of care have frayed or failed. This understanding draws on medical and feminist anthropological traditions that conceptualise care not as a coherent system but as something pieced together through fragile, improvised, and often incomplete arrangements (Graham & Thrift, 2007; Lawson, 2007; Livingston, 2012). A care-patch is one such arrangement: a sociotechnical arrangement of *containment* that helps stabilise individuals at moments of vulnerability, sustaining them until the relational and structural conditions necessary for a more enduring *holding* environment can emerge.

In psychoanalytic traditions, *containment* and *holding* refer to relational conditions that allow distress to be expressed without retaliation or premature resolution (Bion, 1962; Klein, 1946; Winnicott, 1965). Later psychoanalytic writing elaborates containment as an intersubjective process that operates between people, often outside explicit awareness. Ogden’s reformulation of the container-contained relationship emphasises that containment emerges through a relational “third”—a field co-produced in interaction that can do psychic work neither party can accomplish alone (Ogden, 1994, 2004). From this perspective, containment is less a stable attribute of an individual than a temporary configuration of relations that makes thought possible.

Recent work in AI-enhanced psychotherapy similarly treats containment and holding as relational effects that can be partially instantiated through technological mediation, emphasising AI’s role as a *third presence* within the therapeutic field. Haber et al. (2024) describe generative AI as an “artificial third”: an additional element that reorganises relational dynamics without reproducing human intersubjectivity. Rather than containing affect in the strong psychoanalytic sense of transforming distress, such systems participate in configuring the conditions under which affect can be articulated, sustained, and returned in manageable form. This framing resonates with Ogden’s account of the third as emergent and situational, while extending it to consider how non-human intermediaries may alter the psychic ecology of interaction.

The analytic question, then, is not whether such arrangements qualify as “therapy,” but what kinds of psychic work they enable. It is in this sense that Lily’s engagement with AI becomes legible. Her nightly interactions with chatbots can be read as efforts to establish a space where affect can be offloaded without being immediately reinterpreted as fault or failure. The chatbot is not best understood as a therapeutic agent, but as a relational surface—a non-retaliatory interface that can receive emotional overflow and return something sufficiently structured to slow escalation.

By serving as a non-retaliatory interface, the chatbot forces us to ask what kind of relational position a machine actually occupies. This question exposes a central tension within the contemporary care crisis: who is ultimately responsible for listening, and has the capacity to safely hold distress become increasingly confined to clinical settings? Lily’s account repeatedly dissolved the boundaries conventionally maintained within modern systems of care. At various moments, the chatbot resembled a confidant, a companion, a therapist, or a digital diary. Yet because it never fully crystallised into any of these roles, it generated an ambiguity that constitutes the defining feature of the AI care-patch.

Friendship is typically organised through a reciprocal ethic of mutual care, whereas psychotherapy relies on institutional and contractual boundaries to provide a form of unidirectional containment that relieves the speaker of any obligation to reciprocate. As both intimate relationships and public care infrastructures come under increasing strain, however, these distinctions become more difficult to sustain. Under such conditions, the AI chatbot emerges as a liminal technical object, combining the immediacy, flexibility, and accessibility of friendship with the asymmetrical containment associated with psychotherapy, situated at the interface where friendship, therapy, and self-help become increasingly porous. This care-patch brings the politics of listening into sharp relief. Its appeal stems from its ability to occupy a space left open by the simultaneous strain on intimate relationships and formal care infrastructures. More broadly, it reveals how technological mediation has become constitutive of therapeutic communication rather than merely a channel through which care is delivered. AI therefore does not represent a rupture with earlier forms of care. Instead, it makes visible a reality that has long been present: therapeutic exchange has always depended upon mediation.

Interpersonal therapy is often imagined as a dyadic relationship between patient and therapist, yet, as Zeavin (2023) shows, it has always been triadic, organised through a patient, a therapist, and a mediating form—from speech and correspondence to platforms and algorithmic interfaces. Human–machine therapy thus extends rather than disrupts existing processes of externalisation. What therapeutic automation makes explicit is the growing redundancy of one element within this triad: functions historically associated with the therapist—listening, responsiveness, and continuity—are increasingly absorbed by the medium itself. As a result, therapeutic engagement becomes oriented less towards another person than towards the interface, producing what Zeavin terms a “doctorless patient” (2023, p. 325).

This development resonates with broader shifts in psychotherapeutic practice over the past half-century. Cognitive Behavioural Therapy and related approaches have increasingly promoted self-directed models in which patients are responsible for monitoring and regulating their own psychological states (Beck, 1979). In parallel, psychological and cognitive models have been translated into artificial intelligence, and back again, producing informatic frameworks for understanding cognition, language, and affect (Gigerenzer & Goldstein, 1996). Since the late 1950s, successive experiments have sought to mechanise, and at times replace, human participation in the therapeutic dyad, from early conversational programs to contemporary algorithmic interventions.

This trajectory reflects a recurring promise of artificial intelligence: to remove the human other from the communicative relationship. It also echoes a long-standing aspiration within psychotherapy itself—to enable individuals to encounter their own thoughts as if they were those of another (Zeavin, 2023). Yet, despite these developments, therapy continues to be commonly imagined as an interpersonal encounter between two subjects, even as this image has become increasingly anachronistic.

Within this teleology, care delivery aims not at sustaining the clinical relationship but at its sublimation into self-care. As psychiatrist Isaac Marks (1991, p. 42) argues, “refining care delivery to the point where self-care becomes possible is often the product of the most sophisticated stage of a science”. By engaging patients as “mediated selves,” such approaches promise to decouple mental health support from the scarcity of specialist labour, lowering costs and expanding access.

However, automation does not merely replace the “other” but reconfigures the therapeutic encounter into what Joseph and Babu (2024, p. 2) term a *Digital Therapeutic Alliance* (DTA). While automated therapies share technical genealogies with earlier teletherapy, their psychological operation is fundamentally distinct. As Joseph and Babu (2024, p. 2) argue, users frequently anthropomorphise these systems, attributing them with “therapist’s acumen” and empathy—a form of positive transference that replicates the bonds of a traditional therapeutic alliance without the presence of a human subjectivity.

Unlike mediated interpersonal therapy, where technology connects two subjects, AI-driven interaction produces a space of perceived emotional safety precisely because the interlocutor is non-human. By functioning as a consistent, non-judgmental confidant, AI enables what Zeavin (2023) terms “auto-intimacy,” allowing users to disclose experiences they might withhold from human others.

Yet the stability that makes such disclosure possible also carries clinical risk. While an AI interface may function as a container for raw affect, not all forms of containment are transformative. Following Winnicott (1965, pp. 80–84), a distinction can be drawn between a “facilitating environment” that supports psychological growth and forms of holding or management that stabilise distress without enabling change. By design, AI systems may lack the otherness or “optimal frustration” (Kohut, 2009, p. 50) that traditionally propels psychic development. One psychoanalyst I interviewed articulated this tension, suggesting that such interactions risk collapsing into a closed circuit: “Talking to an AI isn’t a conversation with another subject,” they noted. “It’s essentially talking to oneself, and I am afraid all it does is feed narcissism.” While AI may reduce fear of judgement, it also forecloses reciprocal challenge and moral weight, raising the risk of “therapeutic misconception,” where algorithmic feedback is mistaken for empathic understanding (Khawaja & Bélisle-Pipon, 2023, p. 1).

Importantly, Lily was well aware of the limitations of these systems. She frequently noted the chatbot’s tendency towards sycophancy, deliberately adjusting her prompts to elicit more critical and objective responses. In this respect, her engagement reflects what Bucher (2018, p. 117) conceptualises as the “algorithmic imaginary”—a practical, everyday awareness of automated systems that limits complete immersion in the interaction. Lily’s approach, therefore, represents a particular, reflexive mode of engagement rather than a uniform pattern of AI use. For some other users facing particular vulnerabilities, interactions with chatbots may become increasingly immersive and emotionally significant, influencing how they seek support and relate to existing social networks (Morrin et al., 2026). Lily, however, never stopped actively searching for human listeners.

While struggling with anxiety and insomnia, Lily was introduced by a friend to a third-sector charity serving Asian migrants and secured a place on the waiting list for free psychotherapy. She requested a therapist who could speak either Mandarin or Cantonese. “I prefer discussing deeply personal matters in my mother tongue,” she explained, “but that’s only part of the issue.” Lily emphasised that her upbringing—as an “unwanted daughter” burdened by expectations of intergenerational mobility, shaped by the afterlives of state population-control policies, and embedded within the lineage-based kinship structures of rural South China—was difficult to fully articulate in English. She was also uncertain whether an English-speaking therapist could readily grasp the social and historical particularities of her experience. This challenge points to a deeper form of structural exclusion underpinning Illouz’s (2008) argument that *emotional competence*—the ability to introspect, interpret, and communicate feelings in ways recognised by dominant cultural and institutional frameworks—functions as a form of cultural capital. For migrants, this capital does not necessarily travel across linguistic and cultural contexts. Even Western-educated young people like Lily, who are familiar with Euro-American understandings of mental health, may find it difficult to render their experiences legible within dominant clinical frameworks.

Large language models partially circumvented these immediate linguistic and cultural frictions. Using AI allowed Lily to move freely between Mandarin and English, as well as across different systems, including DeepSeek, ChatGPT, and Gemini. She viewed each platform as possessing distinct strengths and weaknesses, making them useful for different purposes. Rather than forcing her to conform to a single therapeutic vocabulary, these systems enabled her to narrate her life across multiple linguistic and cultural registers, giving her a greater sense of agency over how her experiences were articulated and understood.

This frictionless cultural translation reveals a broader implication regarding the structural organisation of care. While Lupton (2026) highlights how uneven access to digital health tools can deepen socio-economic inequalities—whereby the Global North and more affluent populations disproportionately benefit from technological innovation—Lily’s experience points to a different form of stratification. Here, inequality is not driven by a lack of digital access, but by the commodification of human attention and expertise. As high-quality psychotherapy increasingly requires substantial economic, social, and cultural resources, automated and digitised forms of care may become the most accessible, and at times the only readily available option for individuals in more precarious social positions, such as Lily.

This form of stratification often operates through waiting, uncertainty, and gradual disengagement. After spending 6 months on the charity’s waiting list without being matched with a therapist, Lily began to recalibrate her own sense of need and question whether she qualified for support at all. “I don’t expect to get a free or low-cost therapist anymore,” she said. “They probably have lots of people who need the service more than I do, people with ‘real’ mental health problems. Maybe I don’t need it anyway. I should rely on myself.” Once again, institutional scarcity was internalised as a moral judgement about deservingness. In the absence of an explicit refusal, Lily gradually came to interpret the lack of support as evidence that her suffering was insufficiently serious to warrant attention. The management of distress became a matter of personal responsibility rather than collective provision. Within this context, the chatbot functioned as a technology of containment, helping her manage emotional distress while illustrating a broader shift whereby structural constraints are increasingly mediated through practices of self-governance (Rose, 2005).

This trajectory points to a potential long-term tension associated with the digital care-patch. While AI can provide temporary support in the absence of accessible care, sustained reliance on these technologies risks depoliticising individual suffering by obscuring the broader socio-political conditions that produce it. Over time, technological forms of support can reshape expectations of what care can and should provide. The implications of automated therapy are therefore not only psychological but also political. As these technologies become integrated into everyday practices of care, they can normalise diminished access to, and investment in, human infrastructures of listening and support. Rather than merely compensating for gaps in existing systems, digital care-patches increasingly become part of how those gaps are managed and understood. The question, then, is whether the care-patch will gradually come to be treated as care itself.

## Conclusion

This article examines the growing use of artificial intelligence for emotional support to explore how individuals in distress seek to be heard, recognised, and validated when access to care is limited. From this perspective, chatbot-mediated care reflects more than technological innovation; it reveals a broader social condition in which listening has become a scarce and unevenly distributed resource. The significance of AI lies less in its therapeutic efficacy than in what its adoption reveals about the contemporary landscape of care: widening gaps between emotional needs and institutional support, between the desire for intimacy and the mechanisms through which it is accessed, and between experiences of distress and available forms of response. Through an ethnographic analysis of these digital encounters, this article shifts attention from whether machines can truly listen to how listening is organised, distributed, and accessed in contemporary society.

Conceptualising chatbots as “care-patches” offers a way beyond treating artificial intelligence either as an autonomous caregiver or as a technological illusion. As care-patches, these systems are embedded within broader gaps in care provision, responding to unmet emotional needs through provisional forms of support. Their appeal derives not only from constant availability but also from the particular mode of listening they offer—one largely detached from the obligations, expectations, and emotional labour that accompany human relationships. The broader viability of this arrangement depends on the micro-affective experience of “digital holding.” While seeking a human listener often involves costs of time, money, reciprocity, or moral obligation, chatbots provide a low-threshold space for emotional disclosure. Their social efficacy stems, in part, from their non-humanity, which reduces the burdens of relational entanglement.

These findings suggest that digital care-patches do more than compensate for deficiencies in care provision; they participate in the reorganisation of care itself. As emotional distress is increasingly channelled through automated infrastructures, care becomes mediated by technical systems that shape access to recognition and support. Understanding AI in mental health therefore requires attention not only to whether technology can simulate empathy, but also to how it reconfigures the conditions, costs, and accessibility of being heard. Emotional automation neither resolves suffering nor transforms the structural conditions that produce it. Instead, it enables individuals to endure and manage those conditions in everyday life. Chatbots emerge both as products of care deficits and as mechanisms through which those deficits become more manageable. Their broader social significance lies in the way they transform listening from a relational practice embedded in social solidarity and public responsibility into a resource that can be technologically provided, scaled, and managed. Although chatbots have yet to replace human listeners, they are already reshaping the infrastructures of listening, influencing not only how people seek to be heard but also what kinds of attention, recognition, and care they come to perceive as possible, accessible, and deserved.

## Data Availability

No datasets were generated or analysed during the current study.
